# Proteomic analysis of preharvest sprouting in rye using two-dimensional electrophoresis and mass spectrometry

**DOI:** 10.1007/s11032-012-9721-z

**Published:** 2012-03-14

**Authors:** Piotr Masojć, Arkadiusz Kosmala

**Affiliations:** 1 West Pomeranian University of Technology, Słowackiego 17, 71-434 Szczecin, Poland; 2 Institute of Plant Genetics, Polish Academy of Sciences, Strzeszyńska 34, 60-479 Poznań, Poland

**Keywords:** *Secale cereale* L., Preharvest sprouting, Proteins, Two-dimensional electrophoresis, Mass spectrometry

## Abstract

Qualitative and quantitative differences were found between two-dimensional electrophoretic spectra of 546 proteins from two bulked samples of mature rye grain representing: (1) 20 recombinant inbred lines extremely resistant to preharvest sprouting and (2) 20 recombinant inbred lines extremely susceptible to preharvest sprouting. Mass spectrometry of resolved proteins showed that four spots specific for PHS susceptibility represented high molecular weight glutenin subunit, glutathione transferase, 16.9 kDa heat-shock protein, and monomeric alpha-amylase inhibitor. Two spots specific for PHS resistance contained cytosolic malate dehydrogenase and functionally unrecognized protein with sequence homology to rubber elongation factor protein. Majority of 14 proteins with at least two-fold higher accumulation level in preharvest sprouting susceptible lines relative to that found in sprouting resistant lines, showed sequence homology to proteins involved in defense mechanisms against biotic and abiotic stresses including oxidative stress, and those taking part in energy supply. Two spots were identified as regulatory proteins from the 14-3-3 family with one molecular form prevailing in sprouting susceptible and another form highly accumulated in sprouting resistant lines. Further study establishing map positions of the revealed structural genes in respect to quantitative trait loci for preharvest sprouting in rye should answer the question on their possible status as candidate genes.

**Electronic supplementary material:**

The online version of this article (doi:10.1007/s11032-012-9721-z) contains supplementary material, which is available to authorized users.

## Introduction

Genomic architecture of resistance to preharvest sprouting (PHS) in cereals proves that this agronomically important trait has a complex genetic basis (Gale et al. 2002). In rye, all seven chromosomes are engaged in PHS control but 5–6 regions containing quantitative trait loci (QTL) on chromosome arms 1RL, 3RS, 3RL, 5RL and 7RS seem to have the strongest impact (Masojć and Milczarski 2009). A method of bidirectional selective genotyping (BSG) allowed to discern three classes of PHS loci including PHSD loci (directional), PHSR loci (resisting) and PHSE loci (enhancing) which reflect a complex network of epistatic interactions (Masojć et al. 2009; Masojć et al. 2011). This molecular marker—based genomic architecture of PHS in rye needs to be enriched with information on the functional genes underlying particular QTLs. One of the possible ways of identifying genes related to PHS is detection of differences in two-dimensional electrophoresis (2-DE) spectra of grain proteins from the sprouting resistant and sprouting susceptible groups of recombinant inbred lines that were earlier used to study genomic architecture of PHS (Masojć et al. 2009).

The presented work, involved: (1) the analyses of seed protein abundance in the bulked seed samples of the resistant and the susceptible rye inbred lines using 2-DE, (2) mass spectrometry (MS) identification of proteins which were differentially accumulated between the analyzed bulks.

## Materials and methods

Two groups of recombinant inbred lines (F_9_ RILs derived from the Ot1-3 × 541 cross) representing opposite extreme tails of the variation range for preharvest sprouting (Masojć et al. 2009) were grown in 2010 in a six-row plots on the experimental field of West Pomeranian University of Technology in Szczecin, Poland. Spikes, bagged prior pollination period to avoid cross-pollination, were collected from mature plants in the end of July. They were stored in dry conditions at room temperature for 5 days. Sprouted kernels were not detected in both resistant and susceptible groups of lines after visual examination. Dry, mature grain hand-threshed from each of 20 sprouting susceptible lines was bulked (1 g per line) and grounded in RetschMM200 mill. Another bulk sample of the grain from 20 lines extremely resistant to sprouting was collected and milled in a similar way. Plant material used in this study assured that the two compared bulks had similar allele composition at loci not linked to PHS, but contained different alleles at loci crucial for developing resistance or susceptibility to PHS. We assumed that this genetic difference will be reflected on the proteome level.

The protocol for proteomic research performed herein, including two-dimensional electrophoresis to analyze differences in protein accumulation level between both bulks of rye grain and mass spectrometry to identify differentially accumulated proteins, was the same as that described in detail by Kosmala et al. (2009). Protein extraction was performed according to the method described by Hurkman and Tanaka (1986) and protein concentration was determined by the using of 2-D Quant Kit (GE Healthcare). In the first dimension, isoelectrofocusing (IEF), 24 cm Immobiline DryStrip gels with linear pH range 4–7 were used to focus the aliquots of proteins extracted from 50 mg of rye flour. In the second dimension (sodium dodecyl sulphate–polyacrylamide gel electrophoresis) the proteins were separated using 13 % polyacrylamide gels (1.5 × 255 × 196 mm). Rainbow™ molecular weight marker (GE Healthcare) was used as a standard to determine molecular weights (MW) of proteins for particular spots. Following electrophoresis the gels were stained with colloidal coomassie brilliant blue G-250, using the modified method of Neuhoff et al. (1988). Total separated protein spots on the gels were scanned by Image scanner III (GE Healthcare) and subjected to Lab scan 6.0 program (GE Healthcare) processing. Spot detection and image analyses (normalization, spot matching, accumulation comparison) were performed with Image Master 2-D Platinum software (GE Healthcare). To compensate for subtle differences in sample loading, gel staining and destaining, the abundance of each protein spot was normalized as a relative volume (% Vol). The % Vol of each spot was automatically calculated by Image Master software as a ratio of the volume of particular spot to the total volume of all the spots present on the gel. The extraction procedure and electrophoretic separation were performed twice, and the % Vol for the spots from the two replicated gels were then used to calculate means, which were used to make comparisons between analyzed rye lines. The protein spots which showed at least twofold differences in protein abundance between two analyzed lines (quantitative analysis) together with protein spots present only in one of the analyzed lines (qualitative analysis) were subjected to MS analyses and identification.

Peptide samples for MS were prepared using the modified method adapted from Shevchenko et al. (1996). Protein spots were excised from the gel and analysed by liquid chromatography coupled to the mass spectrometer in the Laboratory of Mass Spectrometry, Institute of Biochemistry and Biophysics, Polish Academy of Sciences. Samples were concentrated and desalted on a RP-C18 pre-column (Waters), and further peptide separation was achieved on a nano-ultra performance liquid chromatography (UPLC) RP-C18 column (Waters, BEH130 C18 column, 75 μm i.d., 250 mm long) of a nanoACQUITY UPLC system, using a 45 min linear acetonitrile gradient. Column outlet was directly coupled to the electrospray ionization (ESI) ion source of Orbitrap type mass spectrometer (thermo), working in the regime of data dependent MS to MS/MS switch. An electrospray voltage of 1.5 kV was used. Raw data files were pre-processed with Mascot Distiller software (version 2.3, matrix science). The obtained peptide masses and fragmentation spectra were matched to the National Center of Biotechnology Information (NCBI) non-redundant database with a *Viridiplantae* filter (730,741 sequences) using the Mascot search engine (Mascot Daemon v. 2.3, Mascot Server v. 2.2.03, matrixscience). The following search parameters were applied: enzyme specificity was set to trypsin, peptide mass tolerance to ±40 ppm and fragment mass tolerance to ±0.8 Da. The protein mass was left as unrestricted, and mass values as monoisotopic with one missed cleavage being allowed. Alkylation of cysteine by carbamidomethylation as fixed, and oxidation of methionine was set as a variable modification. Protein identification was performed using the Mascot search probability based Mows score. Ions score was −10* log (*P*), where *P* was the probability that the observed match was a random event. Mascot defined threshold which indicated identity or extensive homology (*P* < 0.05) was 40 or less, therefore ion score 40 was taken as a threshold for analysis. The proteins with the highest multidimensional protein identification technology (MudPIT) scores were selected.

## Results

**Fig. 1:**
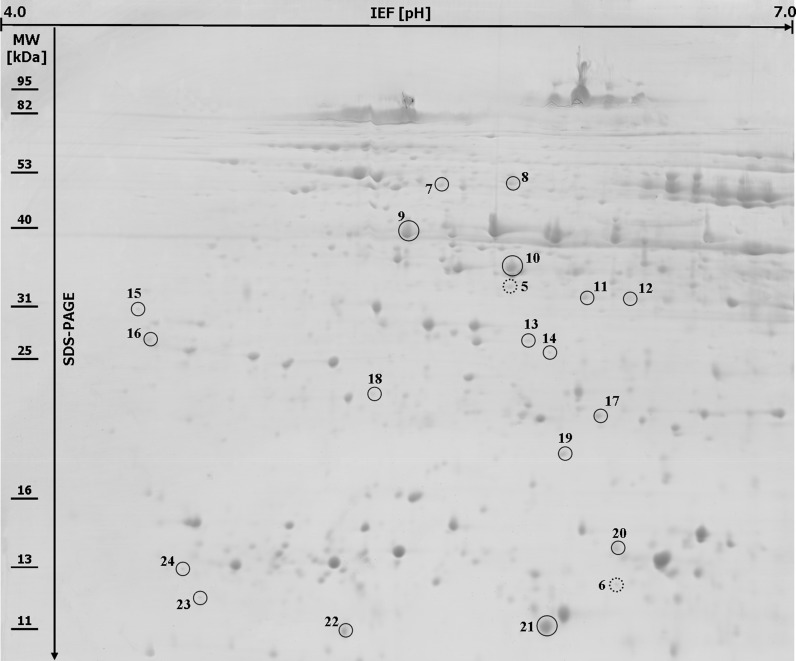
2-DE protein map of mature rye grain from 20 recombinant i*nbred lines* resistant to pre-harvest sprouting. Two protein spots (no. 5 and 6) present only in the PHS resistant lines are* circled* with the *broken lines* and 18 protein spots (no. 7–24) with at least twofold difference in protein abundance between the resistant and susceptible plants are* circled* with the* solid lines* on the gel. Molecular weight (MW) and pH scales are shown

**Fig. 2:**
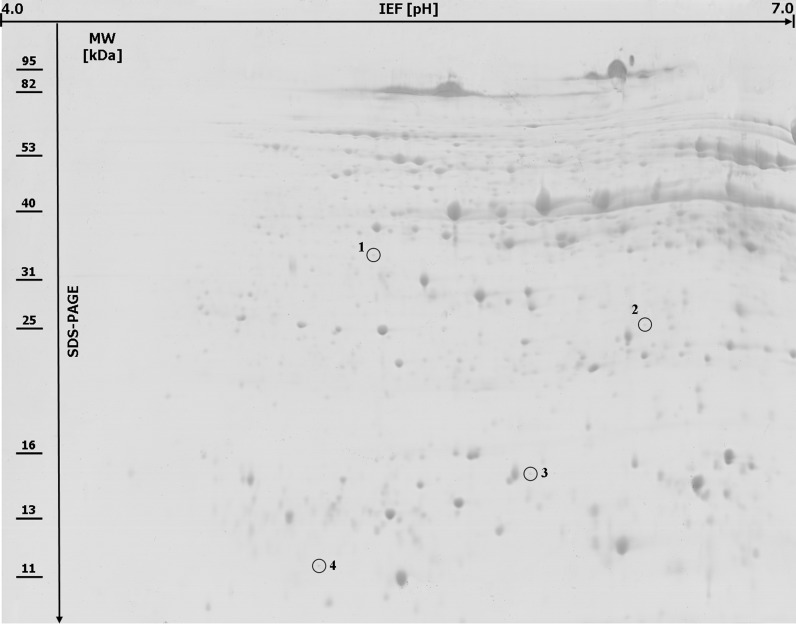
2-DE protein map of mature rye grain from 20 recombinant* inbred lines* susceptible to pre-harvest sprouting. Four protein spots (no. 1–4) present only in the susceptible plants are* circled* with the* solid lines* on the gel

Protein extracts were preliminary analyzed by the use of pH 3–10 and then both 4–7, and 6–9 linear strips for IEF step. Finally, after preliminary results (data not shown), pH 4–7 range was selected as the standard condition for resolving the proteins to achieve the best compromise between the number and resolution of the separated spots. In fact, all the 2-DE patterns within pH 4–7 range were shown to be well-resolved protein maps. Only the spots which were detected within two replicate gels were included into the analyses. Based on this criterion 542 protein spots were reproducibly selected by Image Master 2-DE *Platinum* software within the gels created for the resistant and 544 for the susceptible lines. The comparative analyses revealed a total of 24 spots that showed qualitative and/or quantitative differences in protein abundance between bulks of PHS resistant and susceptible rye lines. The molecular weights of the selected protein spots ranged from 11 to 52 kDa (Fig. 1, 2). Two protein spots (no. 5 and 6) were specific for the sprouting resistant RILs (Fig. 1) and four other protein spots (no. 1, 2, 3 and 4) for the sprouting susceptible RILs (Fig. 2). Eighteen protein spots revealed quantitative differences between analyzed groups of lines (Fig. 1), including four spots with significantly higher protein abundance in the bulk of PHS resistant lines (spots no. 16, 19, 21 and 23) and 14 spots with significantly higher abundance in the bulk of PHS susceptible lines (spots no. 7, 8, 9, 10, 11, 12, 13, 14, 15, 17, 18, 20, 22 and 24).

All the selected protein spots were subjected to mass spectrometry analysis and identification (online resource 1). In most cases the selected proteins derived from rye grain were identified as homologues of proteins from related plant species (Table 1). For protein spots no. 7 and 11 no corresponding amino acid sequence was found in database. They can be considered as “hypothetical proteins” for they represent an open reading frame as revealed by the Mascot software. Proteins present in different amount in grain of sprouting resistant and sprouting susceptible lines belonged to various functional groups. Predominantly, higher accumulation level of proteins taking part in defence mechanisms against biotic and/or abiotic stresses (spots no. 3, 4, 9, 10, 14, 22) including those reducing oxidative stress (spots no. 2, 17, 20) coincided with PHS susceptibility. Also a group of proteins engaged in energy supply showed higher accumulation level in PHS susceptible lines (spots no. 8, 12, 13, 15, 18), similarly as one form of high molecular weight glutenin subunit (spot no. 1) representing seed storage proteins. A single spot (no. 5) identified as cytosolic malate dehydrogenase (MDH) was characteristic for PHS resistant phenotype, together with not well functionally characterized protein (spot no. 6) showing partial homology to rubber elongation factor from distant species of *Hevea brasiliensis.* Two other molecular forms of this protein (spots no. 19 and 23) were present in higher amount in PHS resistant lines, similarly to proteins with sequence homology to dimeric alpha-amylase inhibitor (spot no. 21) and 14-3-3-like protein B (spot no. 16). Proteins, functionally and/or structurally similar to those mentioned above, represented by spots no. 4 (monomeric alpha-amylase inhibitor), 15 (14-3-3 regulatory protein) and 24 (rubber elongation factor) showed higher accumulation level in PHS susceptible lines.

**Table 1:** The results of MS analysis performed on the selected proteins from mature grain of preharvest sprouting resistant and susceptible lines of *Secale cereale* L.

Spot no.^a^	Accession^b^	Identified protein^c^	Cell pathway/molecular function	Score^d^	Coverage ( %)^e^	Protein abundance^f^
1	CAA43331	High molecular weight glutenin subunit (*Triticum aestivum*)	Storage protein	381	9	Present only in SL
2	AAL73394	Glutathione transferase (*Hordeum vulgare*)	Antioxidant and ROS-scavenger pathway	259	12	Present only in SL
3	CAM96540	16.9 kDa heat-shock protein (*Aegilops kotschyi*)	Molecular chaperone/defense	297	29	Present only in SL
4	ACQ83847	Monomeric alpha-amylase inhibitor (*Triticum turgidum*)	Starch metabolizm/defense	146	21	Present only in SL
5	AAT64932	Malate dehydrogenase, cytosolic (*Triticum aestivum*)	Energy metabolism (the citric acid cycle)	461	22	Present only in RL
6	P15252	Rubber elongation factor protein (*Hevea brasiliensis*)	Here ambiguous function	93	7	Present only in RL
7	NP_001059082	Os07g0188800 (*Oryza sativa*)	Unknown	534	15	2.0 × higher in SL
8	P12862	ATP synthase subunit alpha, mitochondrial (*Triticum aestivum*)	Energy metabolism (oxidative phosphorylation)	1566	46	2.1 × higher in SL
9	Q41593	Serpin-Z1A (*Triticum aestivum*)	Protease inhibitor/defense	1243	25	2.8 × higher in SL
10	P93693	Serpin-Z1B (*Triticum aestivum*)	Protease inhibitor/defense	634	19	2.0 × higher in SL
11	NP_001049134	Os03g0175600 (*Oryza sativa*)	Unknown	343	18	2.0 × higher in SL
12	T06212	Glucose and ribitol dehydrogenase homolog (*Hordeum vulgare*)	Carbohydrate metabolizm/energy metabolism	129	11	2.5 × higher in SL
13	Q9SNX2	Phosphoglucomutase, cytosolic (*Bromus inermis*)	Energy metabolism (glycolysis)	138	5	2.1 × higher in SL
14	BAA02948	Tritin (ribosome-inactivating protein) (*Triticum aestivum*)	Protein synthesis/defense	124	9	2.0 × higher in SL
15	CAA74592	14-3-3 protein (*Hordeum vulgare*)	Other protein activity and targeting/energy metabolism	727	36	2.0 × higher in SL
16	Q43470	14-3-3-like protein B (*Hordeum vulgare*)	Other protein activity and targeting/energy metabolism	786	37	2.2 × higher in RL
17	ACV89491	Dehydroascorbate reductase (*Triticum aestivum*)	Antioxidant and ROS-scavenger pathway	815	63	2.0 × higher in SL
18	P46226	Triose phosphate isomerase, cytosolic (*Secale cereale*)	Energy metabolism (glycolysis)	318	29	2.0 × higher in SL
19	P15252	Rubber elongation factor protein (*Hevea brasiliensis*)	Here ambiguous function	82	7	3.0 × higher in RL
20	ACO90194	Superoxide dismutase (*Triticum aestivum*)	Antioxidant and ROS-scavenger pathway	1134	28	2.0 × higher in SL
21	P01085	Dimeric alpha-amylase inhibitor (*Triticum aestivum*)	Starch metabolizm/defense	237	43	3.0 × higher in RL
22	AAS78780	Vacuolar defense protein (*Triticum aestivum*)	Defense	126	13	3.8 × higher in SL
23	P15252	Rubber elongation factor protein (*Hevea brasiliensis*)	Here ambiguous function	101	7	2.3 × higher in RL
24	P15252	Rubber elongation factor protein (*Hevea brasiliensis*)	Here ambiguous function	99	7	3.4 × higher in SL

*ATP* adenosine triphosphate, *RL* resistant lines, *SL* susceptible lines, *ROS* reactive oxygen species

^a^Spot numbering was the same as on Fig. 1 and  2

^b^Database accession (according to NCBInr) of a homologous protein

^c^Homologous protein and organism from which it originates

^d^Mascot MudPIT (multidimensional protein identification technology) score

^e^Amino acid sequence coverage for the identified proteins; amino acid sequences were shown on Fig. S1

^f^Protein abundance was calculated using the mean of relative volumes (% Vol) of two replicates of particular protein spots

## Discussion

One of the main disadvantages of 2-DE is that entire proteomes cannot be visualized in a single gel. Cellular protein populations are diversified with respect of physical properties, making it difficult to collect a complete representation of the proteome in a given extraction and separation procedure. The method of protein sample preparation according to Hurkman and Tanaka (1986), used in the current paper, was shown earlier to be one of the most efficient ways to obtain high quality 2-DE gels with low background staining (e.g. Kosmala et al. 2009).

The fact that only 24 out of 546 distinct spots of 2-DE patterns showed consistent difference in protein accumulation level among PHS resistant and PHS susceptible groups of lines proves high specificity of the performed test, which was gained by applying well defined genetic material. The two groups of recombinant inbred lines were developed by selection of extreme phenotypes in respect to PHS from the population of 5,000 plants representing F_2_ generation of the cross between sprouting resistant (Ot1-3) and sprouting susceptible (541) inbred lines (Masojć et al. 2009). Selection for extreme PHS phenotypes was later carried out in each generation of inbreeding up to a F_7_ progeny. A group of 20 RILs each representing extremely high level of sprouting resistance and a group of 20 PHS susceptible RILs contain a sufficient number of distinct genotypes originating from a single cross, to produce two bulked samples of rye grain with similar genetic background and highly diversified allelic composition at the PHS controlling loci, as shown by their molecular markers (Masojć et al. 2009). Both PHS resistant and PHS susceptible groups of RILs harvested in the end of July 2010 showed no external signs of sprouting. This allowed to compare protein composition in two samples of dry mature grain developing in the same weather conditions that achieved two different physiological stages—grain from PHS resistant RILs attained a long term dormancy whereas that from PHS susceptible RILs was ready to germinate already at the moment of harvest. It is therefore highly probable that differences in the protein composition specified by 2-DE in bulks of dry mature grain represent at least a part of molecular mechanisms leading to extremely different predispositions for preharvest sprouting.

First conclusion which can be drawn from the obtained results is that PHS susceptible grain contains a higher number of proteins with increased accumulation (18 spots) than PHS resistant grain (6 spots). Among six proteins more abundant in PHS resistant grain, three forms represent protein showing partial similarity to the rubber elongation factor from *Hevea brasiliensis*. The fourth spot representing this protein is more abundant in PHS susceptible group. It is not surprising for 2-DE that specific protein like rubber elongation factor can be represented by more than one spot. There are numerous reasons for multiple spots for a given protein including multi-gene families, allozymes, post-translational modifications (phosphorylation, methylation and glycosylation), but also presence of different signals and targeting sequences, in vivo proteolysis or in vitro protein degradation during sample preparation. A class of regulatory proteins was represented in our study by 14-3-3 family (spots no. 15 and 16), differentially accumulated in the compared grain samples. Schultz et al. (1998) revealed interactions between 14-3-3 and VP1 and EmBP1 regulatory proteins from ABA signalling pathways. It is thus possible that 14-3-3 proteins play a role in a complex genetic mechanism of PHS control. Further study aimed at establishing map positions of all identified structural genes in respect to QTLs for PHS should answer the question on their possible status as candidate genes.

Results obtained in rye can be confronted with proteome and transcriptome analysis of grain from PHS resistant and PHS susceptible wheat and rice (Kamal et al. 2009; Qin et al. 2010). Genes for energy supply were upregulated in mature grain of PHS susceptible rice in relation to sprouting resistant line, what supports results obtained in our study. However, in wheat and rice higher accumulation of proteins related to biotic and abiotic stresses was observed in PHS resistant variety. Additionally, particular proteins coinciding with PHS in wheat, rice and rye were different. Therefore, it seems that patterns of genes expression and protein profiles in grain with different predisposition to PHS are species specific and reflect natural selection and breeding having individual history for each crop.

## Electronic supplementary material

Below is the link to the electronic supplementary material.

Supplementary material 1 (PDF 84 kb)
