# Supplementary material for: Proteomic analysis of preharvest sprouting in rye using two-dimensional electrophoresis and mass spectrometry
Source: Mol Breed. 2012 Mar 14;30(3):1355–61. doi: 10.1007/s11032-012-9721-z (PMC3460173; doi:10.1007/s11032-012-9721-z)
Supplement: Supplementary file 1 — Supplementary material 1 (PDF 84 kb) [file 11032_2012_9721_MOESM1_ESM.pdf]

Supplementary Fig. S1. Amino acid sequence of the identified homologous proteins (matched peptides derived from rye are shown in **Red Bold**)

1. **high molecular weight glutenin subunit (*Triticum aestivum*)**

1 MTKRLVLFAA VVVALVALTA AEGEASGQLQ CER**ELQEHSL K**ACR**QVVDQQ**

51 **LR**DVSPECQP VGGGPVAR**QY EQQVVVPPK**G GSFYPGETTP PQQLQQSILW

101 GIPALLRRYY LSVTSPQQVS YYPGQASSQR PGQGQQPGQG QQEYYLTSPQ

151 QSGQWQQPGQ GQAGYYPTSP QQSGQEQPGY YPTSPWQPEQ LQQPTQGQQR

201 **QQPGQGQQLR** QGQQGQQSGQ GQPRYYPTSS QQPGQLQQLA QGQQGQQPER

251 **GQQGQQSGQG QQLGQGQQGQ QPGQK**QQSGQ GQQGYYPISP QQLGQGQQSG

301 QGQLGYYPTS PQQSGQGQSG YYPTSAQQPG QLQQSTQEQQ LGQEQQDQQS

351 GQGRQGQQSG QR**QQDQQSGQ GQQPGQR**QPG YYSTSPQQLG QGQPRYYPTS

401 PQQPGQEQQP RQLQQPEQGQ QGQQPEQGQQ GQQPGQGEQG QQPGQGQQGQ

451 QPGQGQPGYY PTSPQQSGQG QPGYYPTSPQ QSGQLQQPAQ GQQPGQEQQG

501 QQPGQGQQGQ QPGQGQQPGQ GQPGYYPTSP QQSGQEQQLE QWQQSGQGQP

551 GHYPTSPLQP GQGQPGYYPT SPQQIGQGQQ PGQLQQPTQG QQGQQPGQGQ

601 QGQQPGQGQQ GQQPGQGQQP GQGQPGYYPT SLQQSGQGQQ PGQWQQPGQG

651 LPGYYPTSSL QPEQGQQGYY PTSQQQPGQG PQPGQWQQSG QGQQGYYPTS

701 PQQSGQGQQP GQWLQPGQWL QSGYYLTSPQ QLGQGQQPRQ WLQPRQGQQG

751 YYPTSPQQSG QGQQLGQGQQ GYYPTSPQQS GQGQQGYDSP YHVSAEHQAA

801 SLKVAKAQQL AAQLPAMCRL EGGDALLASQ

2. **glutathione transferase (*Hordeum vulgare*)**

1 MAPVK**VFGPA MSTNVAR**VLV CLEEVGAEYE VVDIDFKAME HK**SPEHLVR**N

51 PFGQIPAFQD GDLLLFESRA IAKYVLRKYK TDEVDLLREG NLKEAAMVDV

101 WTEVDAHTYN PALSPIVYEC LINPLMRGLP TNQTVVDESL EKLK**KVLEVY**

151 **EAR**LSQHKYL AGDFVSFADL NHFPYTFYFM ATPHAALFDS YPHVKAWWES

201 LMARPAIKKL AAQMVPKKP

3. **16.9 kDa heat-shock protein (*Aegilops kotschyi*)**

1 MSIVRRTNVL DPFADLWADP FDTFRSIVPA ISGGTSEK**AA FANAR**VDWK**E**

51 **TPEAHVFK**AD LPGVKKEEVK **VEVEDGNVLV VSGER**TKEKE DKNHKWHRVE

101 RSSGKFVRRF RLPEDAMVEE VK**AGLENGVL TVTVPK**TEVK KPEVKAIQIS

151 G

4. **monomeric alpha-amylase inhibitor (*Triticum turgidum*)**

1 MWMKTVFWGL LLFMLVATTM AVEYGARSHN SGPWSWCDPA TGYK**VSALTG**

51 **CR**AMVK**LQCM GSQVPEAVLR** DCCQQLADIN NEWCRCGDLS SMLRSVYQEL

101 GVREGKEVLP GCRKEVMK**LT AASVPEVCK**V PIPNPSGDGA GVCYWAAYPD

151 V

5. **malate dehydrogenase, cytosolic (*Triticum aestivum*)**

1 MAAKEPMRVL VTGAAGQIGY ALVPMIARGV MLGADQPVIL HMLDIEFAAE

51 ALKGVKMELI DAAFPLLKGV VATTDVVEAC TGVNVAVMVG GFPRKEGMER

101 KDVMTKNVSI YK**AQASALEA HAAPNCKVLV VANPANTNAL ILK**EFAPSIP

151 EKNISCLTRL DHNR**ALGQIS ERLGVQVSDV K**NAIIWGNHS SSQYPDVNHA

201 TVKTPSGEKP VRELVQDDEW LNGEFIATVQ QRGAAIIKAR K**LSSALSAAS**

251 **SACDHIR**DWV LGTAEGTFVS MGVYSDGSYG VPAGLIYSFP VTCSGGEWTI

301 VQGLPIDEFS RKK**MDATAQE LSEEK**ALAYS CLA

6. **rubber elongation factor protein (*Hevea brasiliensis*)**

1 MAEDEDNQQG QGEGLKYLGF VQDAATYAVT TFSNVYLFAK DKSGPLQPGV

51 DIIEGPVKNV AVPLYNRFSY IPNGALKFVD STVVASVTII DRSLPPIVK**D**
101 **ASIQVVSAIR** AAPEAARSLA SSLPGQTKIL AKVFYGEN

7. **Os07g0188800 (*Oryza sativa*)**

1 MLRAALLRSG SGLRRPPMAA PLSTAAAASW LSDSASSPPR VRLLIGGEFV
51 ESRADEHVDV TNPATQEVVS R**IPLTTADEF R**AAVDAARTA FPGWRNTPVT
101 TRQRIMLK**YQ ELIR**ANMDK**L AENITTEQGK** TLK**DAWGDVF R**GLEVVEHAC
151 GMGTLQMGEY VSNVSNGIDT FSIREPLGVC AGICPFNFPA MIPLWMFPIA
201 VTCGNTFVLK PSEKDPGAAM MLAELAMEAG LPKGVLNIVH GTHDVVNNIC
251 DDEDIKAVSF VGSNIAGMHI YSRASAKGKR VQSNMGAK**NH AIILPDADR**D
301 ATLNALIAAG FGAAGQRCMA LSTAVFVGGS EPWEDELVKR ASSLVVNSGM
351 ASDADLGPVI SKQAKERICK LIQSGADNGA RVLLDGRDIV VPNFENGNFV
401 GPTLLADVKS EMECYKEEIF GPVLLLMKAE SLDDAIQIVN RNK**YGNGASI**
451 **FTTSGVSAR**K FQTDIEAGQV GINVPIPVPL PFFSFTGSK**A SFAGDLNFYG**
501 **KAGVQFFTQI K**TVTQQWKES PAQRVSLSMP TSQK

8. **ATP synthase subunit alpha, mitochondrial (*Triticum aestivum*)**

1 **MEFSPRAAEL TTLLESRMTN FYTNFQVDEI GRVVSVGDGI ARVYGLNEIQ**
51 **AGEMVEFASG VK**GIALNLEN ENVGIVVFGS DTAIKEGDLV K**RTGSIVDVP**
101 **AGK**AMLGR**VV DALGVPIDGK GALSDHER**RR VEVK**APGIIE RKSVHEPMQT**
151 **GLKAVDSLVP IGR**GQRELII GDRQTGK**TAI AIDTILNQK**Q MNSR**GTNESE**
201 **TLYCVYVAIG QK**RSTVAQLV QILSEANALE YSILVAATAS DPAPLQFLAP
251 YSGCAMGEYF R**DNGMHALII YDDLSK**QAVA YR**QMSLLLR**R PPGREAFPGD
301 VFYLHSRLLE RAAKRSDQTG AGSSTALPVI ETQAGDVSAY IPTNVISITD
351 GQICLETDVF YR**GIRPAINV GLSVSRVGSA AQLK**AMKQVC GSSK**LELAQY**
401 **R**EVAAFAQFG SDLDAASQAL LNRGARLTEV PKQPQYEPLP IEK**QIVVIYA**
451 **AVNGFCDR**MP LDRISQYEK**A ILSTINPELQ K**SFLEK**GGLT NER**KMEPDAS
501 LKESTLPYL

9. **serpin-Z1A (*Triticum aestivum*)**

1 M**ATTLATDVR LSIAHQTR**FA LR**LASTISSN PK**SAASNAAF SPVSLYSALS
51 LLAAGAGSAT RDQLVATLGT GKVEGLHALA EQVVQFVLAD ASSTGGSACR
101 FANGVFVDAS LLLKPSFQEI AVCKYKAETQ SVDFQTK**AAE VTTQVNSWVE**
151 **K**VTSGR**IKDI LPPGSIDNTT KLVLANALYF K**GAWTEQFDS YGTKNDYFYL
201 LDGSSVQTPF MSSMDDQYLL SSDGLKVLKL PYKQGGDNRQ FFMYILLPEA
251 PGGLSSLAEK **LSAEPDFLER** HIPRQRVALR QFKLPKFKIS FGIEASDLLK
301 CLGLQLPFGD EADFSEMVDS LMPQGLR**VSS VFHQAFVEVN EQGTEAAAST**
351 **AIK**MVLQQAR PPSVMDFIAD HPFLFLVRED ISGVVLFMGH VVNPLLSS

10. **serpin-Z1B (*Triticum aestivum*)**

1 MATTLATDVR **LSIAHQTR**FA LR**LASTISSN PK**SAASNAAF SPVSLHSALS
51 LLAAGAGSAT RDQLVATLGT GEVEGGHALA EQVVQFVLAD ASSAGGPRVA
101 FANGVFVDAS LLLKPSFQEL AVCKYKAETQ SVDFQTK**AAE VTTQVNSWVE**
151 **K**VTSGRIKNI LPSGSVDNTT K**LVLANALYF K**GAWTDQFDS YGTKNDYFYL
201 LDGSSVQTPF MSSMDDDQYI SSSDGLKVLK LPYKQGGDNR QFSMYILLPE
251 APGGLSSLAE K**LSAEPDFLE R**HIPRQRVAI RQFKLPKFKI SFGMEASDLL
301 KCLGLQLPFS DEADFSEMVD SPMPQGLR**VS SVFHQAFVEV NEQGTEAAAS**
351 **TAIK**MVPQQA RPPSVMDFIA DHPFLFLLRE DISGVVLFMG HVVNPLLSS

11. **Os03g0175600 (*Oryza sativa*)**

1 MATAASFRPE AARSPPAVQP PAPPLSKFK**V ALCQLSVTAD K**ARNIARARE
51 AIEAAAAGGA KLVLLPEIWN GPYSNDSFPE YAEDIEAGGD AAPSFSMMSE
101 VAR**SLQITLV GGSISER**SGN KLYNTCCVFG SDGELKGKHR KIHLFDIDIP
151 GKITFKESK**T LTAGQDLTVV DTDVGR**IGIG ICYDIRFQEL AMLYAARGAH
201 LLCYPGAFNM TTGPLHWELL QRAR**AADNQL FVATCAPAR**D TSAGYIAWGH

251 STLVGPFGEV IATAEHEETT IMAEIDYSLI DQRRQFLPLQ YQRRGDLYQL
301 VDVQRSGSDE

12. **glucose and ribitol dehydrogenase homolog (*Hordeum vulgare*)**

1 MASQKFPPQQ QDCQPGKEHA MDPRPEAIIK NYKSANKLQG K**VALVTGGDS**
51 **GIGR**AVCLCL ALEGATVNFT YVKGHEDKDA EETLQALRDI KSRTGAGEPK
101 **ALSGDLGYEE NCR**RVVEEVA NAHGGRVDIL VNNAAEQYVR PCITEITEQD
151 LERVFRTNIF SYFLMTKFAV KHMGPGSSII NTTSVNAYKG NATLLDYTAT
201 KGAIVAFTR**A LSMQLAEK**GI RVNGVAPGPI WTPLIPASFP EEKVKQFGSE
251 VPMKRAGQPS EVAPSFVFLA SEQDSSYISG QILHPNGGTI VNS

13. **phosphoglucomutase, cytosolic (*Bromus inermis*)**

1 MVFSVAKKDT TPYEGQKPGT SGLRKKVTVF QQPHYLANFV QSTFNALPAE
51 EVKGATIVVS GDGRYFSKDA VQIIAKMAAA NGVRRVWVGQ GSLLSTPAVS
101 AIIRERIAAD GSKATGGFIL TASHNPGGPT EDFGIKYNMG NGGPAPESVT
151 DKIFSNTKTI SEYLIAEDLP DVDISVIGVT TFTGPEGPFD VDVFDSATEY
201 VKLMKTIFDF ESIKKLLASP KFSFCFDGMH GVAGAYAKRI FVDELGASES
251 SLLNCVPKED FGGGHPDPNL TYAKELVDRM GLGKTSNVEP PEFGAAADGD
301 ADRNMILGKR FFVTPSDSVA IIAANAVQSI PYFASGLKGV ARSMPTSAAL
351 DVVAKNLNLK FFEVPTGWKF FGNLMDAGMC SVCGEESFGT GSDHIREKDG
401 IWAVLAWLSI LAYKNKDNLG GDKLVTVENI VLQHWGIYGR HYYTR**YDYEN**
451 **VDAEAAK**ELM ANLVK**MQSSL SDVNK**LIKEI QPNVADVVSA DEFEYTDPVD
501 GSVSKHQGIR YLFGDGSRLV FR**LSGTGSVG ATIR**IYIEQY EKDSSKTGRE
551 SSDALSPLVD VALKLSKIQE LTGRSAPTVI T

14. **tritin (*Triticum aestivum*)**

1 MAKNVDKPLF TATFNVQASS ADYVTFINGI RNKLRNPGHS SHNRPVLPPI
51 EPNVPPSRWF HIVLK**TSPAS TGLTLATR**AD NLYWEGFKSS DGTWWELTPG
101 LIPGATHVGF GGTYRDLLGD TDKLTNVALG R**QQMADAVTA LYGR**TKADKT
151 SGPKQQQARE AVTTLLLMVH EATRFQTVSG FVAGVLHPKE KKSGKIGNEM
201 KAQVNGWQDL SEALLKTDAN APPGKAPAKF TPIEKMGVRT AEQAAATLGI
251 LLFVQVPGGM TVAQALELFH KSGGK

15. **14-3-3 protein (*Hordeum vulgare*)**

1 MSAPGELSR**E ENVYMAKLAE QAERYEEMVE FMEK**VAK**TVD SEELTVEER**N
51 LLSVAYKNVI GARRASWR**II SSIEQKEESR** GNEDRVTLIK EYR**GKIETEL**
101 **SK**ICDGILK**L LETHLVPSST APESK**VFYLK MKGDYYRYLA EFKSGPER**KD**
151 **AAENTMVAYK** AAQDIALAEL APTHPIRLGL ALNFSVFYYE ILNSPDRACN
201 LAKQAFDEAI SELDTLSEES YK**DSTLIMQL LR**DNLTLWTS DITEDTAEEE
251 IREAPKHDSS EGQ

16. **14-3-3-like protein B (*Hordeum vulgare*)**

1 MAQPAELSR**E ENVYMAKLAE QAERYEEMVE FMEK**VAK**TVD SEELTVEER**N
51 LLSVAYKNVI GARRASWR**II SSIEQK**EESR GNEDRVTLIK DYR**GKIEVEL**
101 **TKICDGILKL LDSHLVPSST APESK**VFYLK MKGDYYRYLA EFKSGTER**KD**
151 **AAENTMVAYK** AAQEIALAEL PPTHPIRLGL ALNFSVFYYE ILNSPDRACD
201 LAKQAFDEAI SELDSLSEES YK**DSTLIMQL LR**DNLTLWTS DISEDAAEEM
251 KDAPKGESGD GQ

17. **dehydroascorbate reductase (*Triticum aestivum*)**

1 MTEVCVK**AAV GHPDTLGDCP FSQRVLLTLE EK**KVPYQMKL IDVSNKADWF
51 LKINPEGK**VP VYNGGDGKWI ADSDVITQVI EEKYPTPSLV TPPEYASVGS**
101 **KIFSTFVTFL K**SKDASDGSE K**ALVDELQAL EEHLKAHGPY INGANISAVD**
151 **LSLAPKLYHL QVALEHFK**GW KVPETLTSVH AYTEALFSRE SFVKTKATK**E**

201 **NLIAGWAPK**V NP.

18. **triosephosphate isomerase (*Secale cereale*)**

1 MGRKFFVGGN WKCNGTVSQV ETIVNTLNAG QIASPDVVEV VVSPPYVFLP
51 TVKDKLRPEI QVAAQNCWVK KGGAFTGEVS AEMLVNLGIP WVILGHSERR
101 **SLLAESSEFV GEKVAYALAQ GLKVIACVGE TLEQREAGST MEVVAEQTK**A
151 IADKIKDWTN VVVAYEPVWA IGTGKVASPA QAQEVHANLR DWLK**TNVSPE**
201 **VAESTRIIYG GSVTGASCK**E LAAQPDVDGF LVGGASLKPE FIDIINAATV
251 KSA

19. **rubber elongation factor protein (*<u>Hevea brasiliensis</u>*)**

1 MAEDEDNQQG QGEGLKYLGF VQDAATYAVT TFSNVYLFAK DKSGPLQPGV
51 DIIEGPVKNV AVPLYNRFSY IPNGALKFVD STVVASVTII DRSLPPIVK**D**
101 **ASIQVVSAIR** AAPEAARSLA SSLPGQTKIL AKVFYGEN

20. **superoxide dismutase (*Triticum aestivum*)**

1 MVK**AVAVLTG SEGVK**GTIFF TQEGEGPTTV TGSVTGLKEG LHGFHVHALG
51 DTTNGCMSTG PHFNPAGHVH GAPEDEIRHA GDLGNVTAGV DGVASINITD
101 CHIPLTGPNS IVAR**AVVVHG DADDLGK**GGH ELSK**STGNAG ARVACGIIGL**
151 **QG**

21. **alpha-amylase inhibitor (*Triticum aestivum*)**

1 SGPWMCYPGQ AFQVPALPAC RPLLR**LQCNG SQVPEAVLR**D CCQQLAHISE
51 WCR**CGALYSM LDSMYK**EHGA QEGQAGTGAF PRCRREVVK**L TAASITAVCR**
101 **LPIVVDASGD GAYVCK**DVAA YPDA

22. **vacuolar defense protein (*Triticum aestivum*)**

1 MAARLALVVA LLCAGAAAAA AQQASNVRAT YHYYRPAQNG WDLGAPAVSA
51 YCSTWDAGKP YSWRSRYGWT AFCGPAGPRG QASCGRCIR**V TNTGTGAQIT**
101 **AR**IVDQCANG GLDLDWDTVF VK**IDTDGMGY QR**GHLIVNYQ FVDCRDNHIN
151 FHGKNETLPA STDAVA

23. **rubber elongation factor protein (*<u>Hevea brasiliensis</u>*)**

1 MAEDEDNQQG QGEGLKYLGF VQDAATYAVT TFSNVYLFAK DKSGPLQPGV
51 DIIEGPVKNV AVPLYNRFSY IPNGALKFVD STVVASVTII DRSLPPIVK**D**
101 **ASIQVVSAIR** AAPEAARSLA SSLPGQTKIL AKVFYGEN

24. **rubber elongation factor protein (*<u>Hevea brasiliensis</u>*)**

1 MAEDEDNQQG QGEGLKYLGF VQDAATYAVT TFSNVYLFAK DKSGPLQPGV
51 DIIEGPVKNV AVPLYNRFSY IPNGALKFVD STVVASVTII DRSLPPIVK**D**
101 **ASIQVVSAIR** AAPEAARSLA SSLPGQTKIL AKVFYGEN
